# Laryngeal Rosai-Dorfman Disease (Sinus Histiocytosis with Massive Lymphadenopathy): A Retrospective Study of 5 Cases

**DOI:** 10.1155/2017/8521818

**Published:** 2017-07-09

**Authors:** Yanyan Niu, Yongjin Li, Jian Wang, Xiaofeng Jin, Dahai Yang, Hong Huo, Wuyi Li

**Affiliations:** Department of ENT, Peking Union Medical College Hospital, Chinese Academy of Medical Sciences, Beijing, China

## Abstract

This study was performed to investigate the clinical manifestations, treatment methods, and prognosis of Rosai-Dorfman disease (RDD) with laryngeal involvement. Five clinical cases of RDD with laryngeal involvement diagnosed between 1986 and 2015 were retrospectively analyzed. The laryngeal lesions of these 5 patients mostly involved the glottis and subglottis, with the main symptoms being a hoarse voice and airway obstruction. In addition, the patients mostly exhibited a unilateral or asymmetric onset that was manifested by a laryngeal submucosal nodular mass. The patients were subjected to a regimen of hormone treatment combined with surgical resection. The median follow-up duration was 101 months (8–384 months). One case was lost, and the remaining 4 subjects are alive with disease. The follow-up examinations revealed that 4 subjects had stable laryngeal conditions, whereas one showed minor progression. RDD with laryngeal involvement is clinically rare and differs considerably from classical RDD in age of onset, gender composition, and extranodal involvement. The regimen of hormone treatment combined with surgical resection can stabilize the patient's general condition and laryngeal lesion. Tracheotomies are recommended for patients with dyspnea. After their conditions stabilize, decannulation can be successfully performed in most cases. This therapeutic regimen generally delivers a good prognosis.

## 1. Introduction

Rosai-Dorfman disease (RDD) was named after Rosai and Dorfman, who were the first to describe the illness in 1969 [1]. In 1972, Rosai and Dorfman [2] performed clinicopathological analyses on 34 patients and named this disease sinus histiocytosis with massive lymphadenopathy (SHML), which is characterized by painless lymphadenopathy, fever, patches of massive proliferation of histiocytes, and the presence of engulfed lymphocytes in the cytoplasm of these histiocytes. RDD is clinically rare and generally involves the lymph nodes; RDD can also present in any extranodal site [3], with the most common sites being the skin and central nervous system [3]. In comparison, RDD with laryngeal involvement is extremely rare, and there are only isolated cases in the literature. In this study, we examined 5 cases of RDD with laryngeal involvement that were treated in our hospital, and we reviewed the literature regarding RDD with laryngeal involvement. We suspect that our analyses may improve the diagnosis and treatment of this rare disease.

## 2. Materials and Methods

Between 1986 and 2015, there were 31 cases of RDD, and the diagnoses were confirmed pathologically in our hospital. Five cases of RDD with laryngeal involvement were retrospectively examined. General information such as gender, disease onset, clinical manifestations, laboratory tests, and pathological information were retrieved from the clinical records. Experienced senior pathologists were invited to further validate the laryngeal pathological sections of the 5 patients. During follow-up visits, the subjects were examined for their survival, previous treatment methods for the laryngeal lesions, and the corresponding efficacy. SPSS 17.0 was used for statistical analyses, and descriptive analysis was employed for general information. The study was approved by the Ethics Committee of Beijing Union Medical College Hospital.

## 3. Results

### 3.1. General Patient Information

The general information for all 5 patients is summarized in Table 1, whereas the clinical features of their larynges are summarized in Table 2. The patients included 3 males and 2 females with a median age of 38 years (27–45 years) at RDD diagnosis. The symptoms of systemic onset mainly included lymphadenopathy in the neck, armpits, and groin as well as laryngeal symptoms (mainly a hoarse voice and airway obstruction). Cases #4 and 5 did not have laryngeal symptoms at onset, but laryngeal symptoms appeared at the time of diagnosis; cases #1–3 did not present any laryngeal symptoms at the time of diagnosis, but the laryngeal involvement and symptoms were confirmed during follow-up. Upon RDD diagnosis, cases #1–3 did not undergo laryngoscopy, and, therefore, no laryngeal lesions were revealed initially. The laryngeal symptoms of these patients occurred 1-2 years after the RDD diagnosis when the laryngeal involvement was established. Cases #4 and 5 were shown to have laryngeal lesions upon laryngoscopy during the first examination. In particular, case #5 was found to have primary symptoms including airway obstruction and a hoarse voice, and laryngeal lesions were immediately identified via laryngoscopy. In addition to the larynx, the nasal cavity (3 cases) and pharyngeal cavity (2 cases) were also commonly involved, followed by the skin (1 case) and salivary gland (1 case).

### 3.2. Laryngeal Manifestations and Pathology

**Figure 1:**
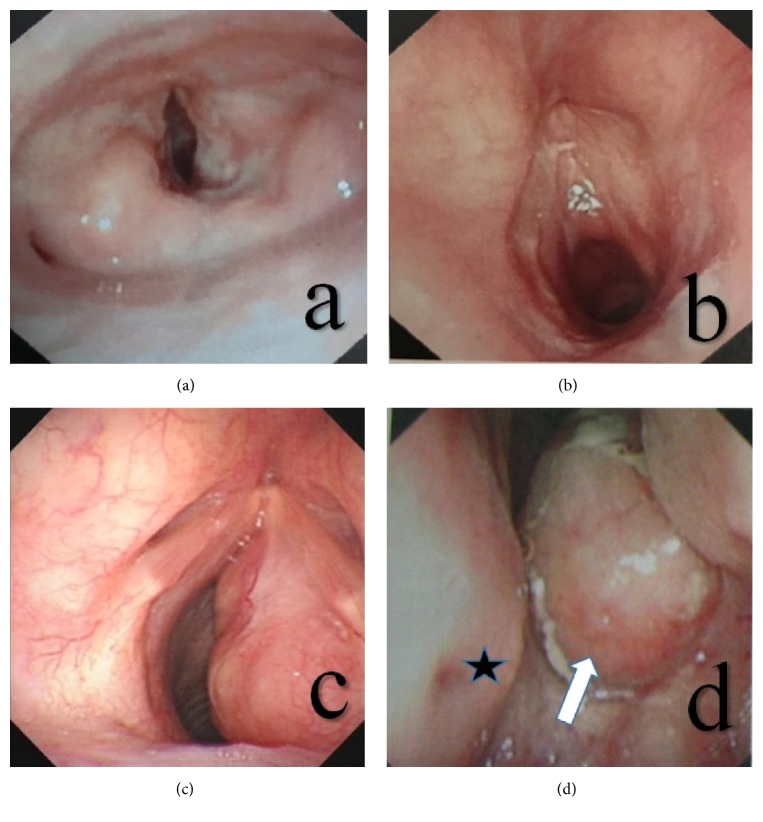
A laryngoscopic view of a classical case of RDD with laryngeal involvement. (a) This picture shows that the nodular lesions with smooth surfaces were mainly present in the right vocal cord and the posterior region of the subglottic area. (b) This picture shows that the nodular lesions with smooth surfaces were mainly present in the right vocal cord and the anterior region of the subglottic area. (c) This picture shows that the nodular, ulcer-free lesions with smooth surfaces were mainly present in the right vocal cord and entire subglottic area. (d) This picture shows the nasopharyngeal manifestation of the same patient in panel (c). The white arrowhead points to nasopharyngeal nodular lesions with smooth mucous membranes; the black star indicates the nasal septum.

**Figure 2:**
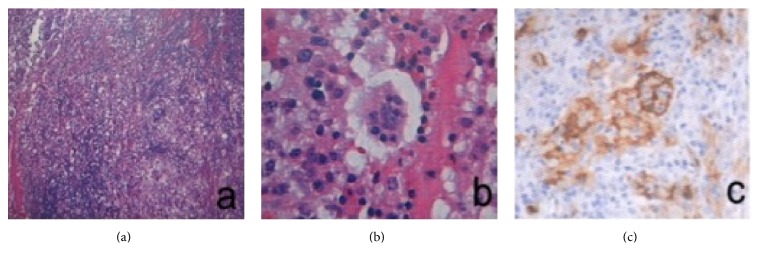
Pathology of a classical case of RDD with laryngeal involvement. (a) HE staining, 60x, the patches of histiocyte proliferation form nodular zones with light staining. (b) HE staining, 300x, classical histiocytes with large nuclei and low mitotic counts; engulfed lymphocytes are present in the cytoplasm. (c) Immunohistochemical analysis revealed that the histiocytes were S-100 positive.

All 5 patients were found to have laryngeal lesions via laryngoscopy, which mostly involved the glottis and subglottis. In particular, the subglottic lesions were more pronounced than the glottic lesions, which often presented as unilateral or asymmetric lesions with the onset of disease and manifested a laryngeal submucosal nodular mass, a tough texture, and resistance to bleeding. In addition, some of the laryngeal tumors were solid and cystic in nature and contained mucus in the lumen (Figure 1). The laryngeal tumors pathologically manifested classical RDD traits (Figure 2), and the lesions were mainly concentrated under the mucosa. At low magnification, lightly stained nodular zones containing patches of histiocytic proliferation were visible, and, under high magnification, classical histiocytes with large nuclei, low mitotic counts, and engulfed lymphocytes in the cytoplasm were visible. Immunohistochemistry revealed cellular expression of the S-100 and CD68 proteins.

### 3.3. Course of Treatment and Outcome

The course of treatment and results of the follow-up visits are summarized in Table 3. All the subjects were treated with adrenal glucocorticoid, with case #4 also receiving cladribine. Two patients who were treated in the early stages of RDD (cases #1 and 2) underwent open surgery and tracheotomies. The other three patients (cases #3–5) all underwent endoscopic surgery to treat the laryngeal lesions. Tumor relapse occurred in cases #3 and #4 after the first endoscopic resection of the tumors; therefore, a secondary endoscopic operation was performed to remove the tumors. After surgery, case #3 developed an aggravated laryngeal condition involving expiratory dyspnea and underwent a tracheotomy 61 months after the diagnosis of RDD. Case #5 received a tracheotomy during the first treatment due to airway obstruction.

**Figure 3:**
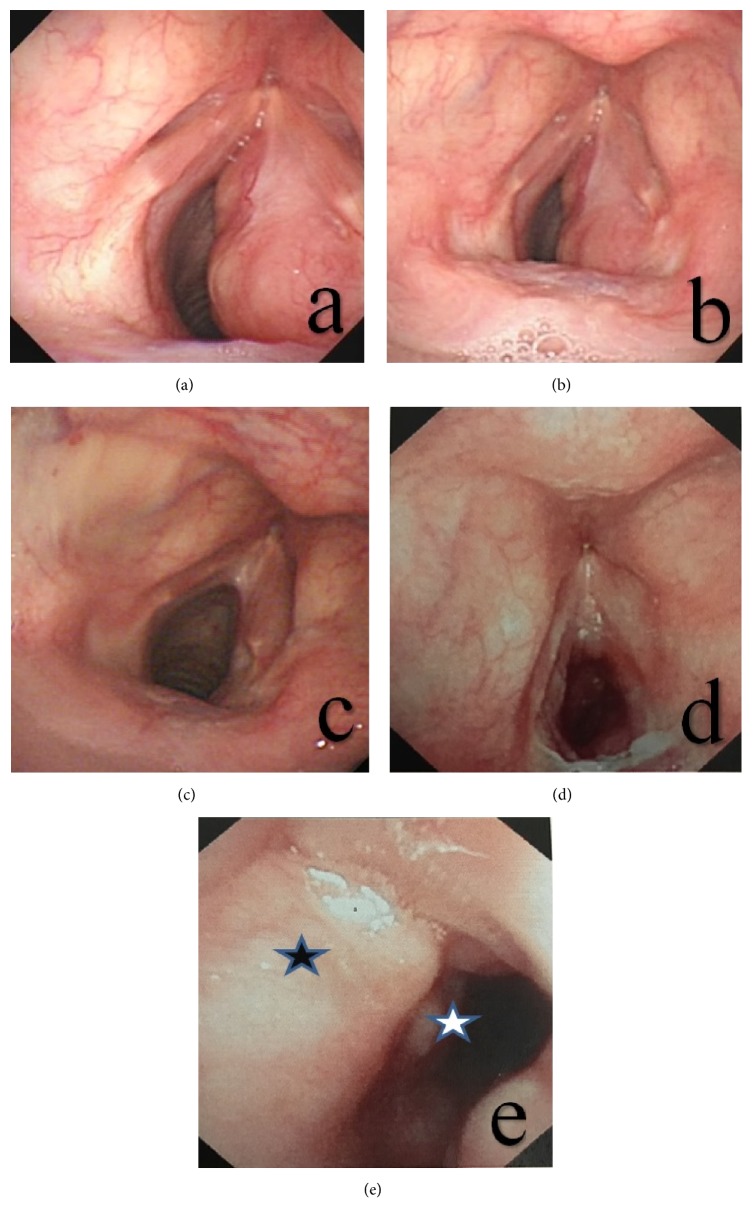
The course of treatment and relevant efficacy for a classical case of RDD with laryngeal involvement. (a) The pretreatment laryngoscopy revealed a normal left vocal cord but involvement in the right vocal cord and subglottic area. The movement of the right vocal cord was restricted. (b) Review after 1 month of prednisone 30 mg × 2 W + cladribine 50 mg × 1 W showed no apparent changes in the laryngeal lesions. (c) A review of the laryngoscopy images generated 6 months after the endoscopic operation revealed that the subglottic tumors had almost disappeared and that the subglottic trachea displayed no apparent abnormalities. (d) A review 3 years after the first endoscopic surgery revealed progression of the lesions in the inferior and anterior regions of the glottis. (e) A review 3 years after the endoscopic surgery revealed that the subglottic lesions were partially obstructing the airway. Black stars indicate the lesions in the anterior region of the glottis, whereas white stars indicate the newly developed subglottic lesions.

The median follow-up duration of the cohort was 101 months (8–384 months). Among the patients, case #2 survived with the disease for the 165 months of follow-up but was later lost to follow-up. Case #3 underwent 2 operations and a treatment regimen with prednisone and cladribine, but the laryngeal lesions still exhibited slow progression. This subject is currently refusing another laryngeal operation and was therefore not decannulated. The remaining subjects all exhibited stable conditions, had no expiratory dyspnea, and were decannulated (Figure 3).

## 4. Discussion

RDD is a rare, nonmalignant type of histiocytosis that occurs mostly in the young and has the main clinical manifestations of giant and painless cervical lymphadenopathy, fever, and an elevated erythrocyte sedimentation rate [4]. The disease has a male : female ratio of approximately 5 : 1 [5] and an extranodal involvement rate of below 50% [6] or approximately 25–43% [7]. The most common sites of extranodal involvement include the skin, soft tissue, central nervous system, and upper respiratory tract [3, 7]. RDD with laryngeal involvement is extremely rare, and less than 30 such cases have been reported in the English literature so far, with most being reports of isolated cases [7]. To our knowledge, this study has the largest cohort size of RDD cases with laryngeal involvement. The main clinical manifestations of RDD with laryngeal involvement include foreign body sensation, hoarse voice, dyspnea, and cough [7]. Examination of the laryngeal symptoms of the 5 patients revealed that, in the early stage of involvement, no apparent laryngeal symptoms were present. Once laryngeal symptoms appeared, the lesions could be identified via laryngoscopy, with the most prominent manifestations being voice alteration and expiratory dyspnea. For RDD patients with laryngeal involvement, upper respiratory lesions are often identified in the nasal cavity and pharynx through examination, suggesting that laryngeal lesions may only represent a portion of the upper respiratory involvement. As such, if the conditions are not promptly controlled, multiple sites in the upper respiratory tract may become involved. Involvement outside of the upper respiratory tract was uncommon in this study, with only 1 case exhibiting skin involvement. The age of onset for the RDD patients with laryngeal involvement in this study was higher than that for common RDD patients. The male : female ratio of the RDD patients with laryngeal involvement was 3 : 2, which is consistent with that of the RDD cases of the digestive system that were reported previously [4]. RDD with laryngeal involvement differs from classical RDD with regard to age, gender composition, and extranodal involvement, which argues that RDD with extranodal involvement is a subtype of RDD that differs from RDD that has only lymph node involvement described in the clinical traits [8].

Despite different clinical manifestations, the following pathological traits are still consistent between RDD with laryngeal involvement and classical RDD: the presence of dark and light colored patches that are visible under low magnification and are formed by nodular areas of distributed histiocytes and dense areas of inflammatory cell infiltration. Under high magnification, the lightly stained area comprises large histiocytes, which often contain engulfed lymphocytes. The extranodal lesions and the lymph node lesions were very similar, but the extranodal lesions showed more obvious fibrosis and fewer histiocytes, and emperipolesis was not common. The immunohistochemistry results suggested that these cells were S-100-positive, CD68-positive, and CD1a-negative; staining with GMS and PAS was negative, thereby excluding infectious diseases, which aids in differentiating RDD from other diseases [9, 10]. Although the etiology of RDD remains unclear, evidence from Paulli et al. suggests that RDD is not a tumor disease. Instead, RDD is more likely to be an inflammatory disease based on a cloning analysis via polymerase chain reaction (PCR) experiments [11].

Currently, there are no ideal treatments for RDD; however, it has been reported that 20% of patients exhibited spontaneous resolution [12]. Adrenal glucocorticoid is a commonly used drug, and 1/3 of RDD patients have been reported to respond to the administration of adrenal glucocorticoid [13]. A previous study reported that a regimen combining vincristine with glucocorticoid achieved an efficacy of 50% [14]. Radiotherapy has also been reported to treat RDD [15]. For RDD lesions with laryngeal involvement, surgical resection is a general recommendation because involvement at this location may cause life-threatening dyspnea. The surgical approach has a relatively low recurrence rate and increases long-term survival [7, 16]. Generally, RDD has a good prognosis, but the presence of laryngeal involvement may threaten the safety of the airway. Therefore, it is recommended that patients with RDD undergo a careful laryngoscopy to exclude laryngeal involvement. For those RDD patients with laryngeal involvement, surgery is the most effective method of treatment and is also essential for diagnosis [17]. This study revealed the following findings: (i) all patients achieved long-term survival, and their laryngeal lesions from RDD were stabilized and (ii) although our treatment could not completely eradicate the disease, none of the patients developed lesions in other important organs, and all were registered as alive with disease. Hence, the RDD patients with laryngeal involvement had good prognoses. Furthermore, the individuals with stable conditions who are free of dyspnea can undergo decannulation and enjoy a relatively good quality of life.

## 5. Conclusion

RDD with laryngeal involvement is clinically rare and differs from classical RDD with regard to the age of onset and gender ratio. The patients who were treated with the regimen involving hormone administration and surgical resection exhibited stable laryngeal conditions. For individuals with dyspnea, tracheotomy should be considered, and once the condition stabilizes, decannulation can be performed. In general, the treatments resulted in good prognoses.

## Figures and Tables

**Table 1:** The general information for the 5 RDD cases with laryngeal involvement.

Case number	Age upon RDD diagnosis (years)	Age upon laryngeal RDD diagnosis (years)	Gender	Onset of systemic symptoms	Sites of lymph node involvement
1	27	28	M	Cervical lymphadenopathy	Neck, axilla
2	34	36	F	Cervical lymphadenopathy	Neck
3	39	41	M	Nasal obstruction and cervical lymphadenopathy	Neck, axilla, groin
4	38	38	M	Cervical lymphadenopathy	Neck, axilla
5	45	45	F	Hoarse voice, suffocation	NA

**Table 2:** The clinical features of larynges for the 5 RDD cases with laryngeal involvement.

Case number	Onset of laryngeal symptoms	Sites of laryngeal involvement	Events leading to discovery of laryngeal involvement	Sites of extranodal involvement
1	Hoarse voice	Glottis and subglottic region	Laryngoscopy due to hoarse voice	Skin
2	NA	Subglottic region	Laryngoscopy due to nasal obstruction	Nasal cavity
3	Hoarse voice	Glottis and subglottic region	Laryngoscopy due to hoarse voice	Nasal cavity, pharynx, submandibular gland
4	NA	Glottis and subglottic region	Routine laryngoscopy	Nasal cavity, pharynx
5	Hoarse voice and suffocation	Glottis and subglottic region	Routine laryngoscopy	NA

**Table 3:** The course of treatment and relevant efficacy for the 5 RDD cases with laryngeal involvement.

Case number	Follow-up (months)	Systemic regimen	Laryngeal treatment strategy	Condition of laryngeal lesions	Outcome
1	384	Prednisone 20 mg × 4 W	Open surgery + tracheotomy	Stable	AWD
2	165	Prednisone 30 mg × 2 W	Open surgery + tracheotomy	Stable	AWD/LFU
3	101	Prednisone 30 mg × 2 W + cladribine 50 mg × 1 W	Endosurgery ×2 + tracheotomy	Slow progress	AWD
4	98	Prednisone 30 mg × 2 W	Endosurgery ×2	Stable	AWD
5	8	Prednisone 30 mg × 2 W	Endosurgery + tracheotomy	Relieved	AWD

Note: AWD, alive with disease; LFU, lost to follow-up.
